# Effect of Leaf Trichomes in Different Species of Cucurbitaceae on Attachment Ability of the Melon Ladybird Beetle *Chnootriba elaterii*

**DOI:** 10.3390/insects13121123

**Published:** 2022-12-05

**Authors:** Valerio Saitta, Manuela Rebora, Silvana Piersanti, Elena Gorb, Stanislav Gorb, Gianandrea Salerno

**Affiliations:** 1 Dipartimento di Scienze Agrarie, Alimentari e Ambientali, University of Perugia, Borgo XX Giugno 74, 06121 Perugia, Italy; 2 Dipartimento di Chimica, Biologia e Biotecnologie, University of Perugia, Via Elce di Sotto 8, 06121 Perugia, Italy; 3 Department of Functional Morphology and Biomechanics, Zoological Institute, Kiel University, Am Botanischen Garten 9, 24098 Kiel, Germany

**Keywords:** friction, adult, larva, pulvilli, claws, plant trichomes, biomechanics

## Abstract

**Simple Summary:**

The investigation of insect attachment ability in relation to plant mechanical barriers can shed light on the different steps driving host plant selection in phytophagous insects and help to better understand the complex antagonistic coevolution between insects and plants. In this context, we investigated the attachment ability of the oligophagous melon ladybird beetle *Chnootriba elaterii* at different developmental stages (adult, larva, eggs) to leaves of several Cucurbitaceae species (watermelon, melon, cucumber, zucchini, pumpkin, squirting cucumber, calabash and loofah) characterized by the presence of glandular and non-glandular trichomes with different characteristics (density, length). We used different techniques (scanning electron microscopy, traction force experiments and centrifugal force tests) to characterize the plant leaf surface and insect attachment devices and measure insect attachment ability to the leaf of the different plant species. Data regarding morphological leaf traits of Cucurbitaceae associated with their resistance against phytophagous insects, combined with data regarding the chemical cues involved in the host plant selection, can help to develop environmentally friendly pest control methods.

**Abstract:**

This study investigates the attachment ability of the oligophagous melon ladybird beetle *Chnootriba elaterii* to leaves of several Cucurbitaceae species. Using cryo-SEM, we described adult and larva tarsal attachment devices and leaf surface structures (glandular and non-glandular trichomes) in *Citrullus lanatus*, *Cucumis melo*, *Cucumis sativus*, *Cucurbita moschata*, *Cucurbita pepo, Ecballium elaterium*, *Lagenaria siceraria* and *Luffa aegyptiaca*. Using traction force experiments and centrifugal force tests, we measured the friction force exerted by females and larvae on plant leaves. We observed that Cucurbitaceae glandular trichomes do not affect insect attachment ability at both developmental stages, suggesting some adaptation of *C. elaterii* to its host plants, while non-glandular trichomes, when they are dense, short and flexible, heavily reduce the attachment ability of both insect stages. When trichomes are dense but stiff, only the larval force is reduced, probably because the larva has a single claw, in contrast to the adult having paired bifid dentate claws. The data on the mechanical interaction of *C. elaterii* at different developmental stages with different Cucurbitaceae species, combined with data on the chemical cues involved in the host plant selection, can help to unravel the complex factors driving the coevolution between an oligophagous insect and its host plant species.

## 1. Introduction

Small insect herbivores face the problems of attachment to plant leaves at every stage of their development. On the other hand, in the long antagonistic evolution between phytophagous insects and plants, the latter have developed different chemical and mechanical defenses to protect themselves from consumption. In response to the high insect attachment ability guaranteed by pads (hairy or smooth) and claws typically located in the tarsus and pretarsus [1], many plants have developed anti-adhesive surfaces (see review in [2]). Plant anti-adhesive features can be represented by cell shape [3], wet surfaces [4], cuticular folds [5], epicuticular wax projections (e.g., [6], see review in [7]) or their combinations. These characteristics inducing surface slipperiness are particularly developed in insect-trapping plants [4,8,9,10]. Trichomes are hair-like protuberances extending from the epidermis, which characterize the surface of many plant species. They can be very different in shape (straight, hooked, branched or unbranched), size, orientation, structure and secretory function [11,12]. Leaf pubescence can play an important role against herbivorous insects [11,13,14,15,16,17]. For instance, the ability of the hooked trichomes of *Phaseolus* plants (Fabaceae) to entrap and kill arthropod species belonging to different insect orders is well known (e.g., [18]; see review in [19]. Another example is the glabrous morph of *Arabidopsis lyrata* (L.) O’Kane and Al-Shehbaz (Brassicaceae) which is subjected to higher damage by phytophagous insects than the morph with trichomes [20]. It has been observed that many plant species respond to insect feeding damage by producing new leaves with an increased density and/or increased number of trichomes (see review in [21]. The effect of leaf pubescence may be different according to the herbivore species [11,22], the trichome type (glandular, with the function to secrete metabolites for the plant or non-glandular) as well as trichomes length and distribution [23]. In general, long-legged sucking insects such as Hemiptera tend to be less deterred by trichomes, and sometimes a positive effect of trichomes in enhancing insect locomotion has been described. This is the case of the generalist stinkbug *Nezara viridula* (L.) (Hemiptera: Pentatomidae), which showed a stronger attachment force on the non-glandular stellate trichomes of *Solanum melongena* L. (Solanaceae) than on smooth glass [24], probably due to the use of claws interlocking to the trichomes and by this improving attachment during pulling. A very good performance on hairy plant surfaces was demonstrated also by the omnivorous mirid bug *Dicyphus errans* (Wolff) (Hemiptera: Miridae) [12] that typically lives on pubescent plants and shows morphological and behavioural adaptations to hairy plants resulting in higher predation, fecundity, and attachment ability, if compared to plants without trichomes. On the opposite, trichomes can have a detrimental effect on species showing a high degree of ventral contact with leaf surfaces such as lepidopteran and sawfly larvae [11,13,22,25,26].

Among Coleoptera, in Coccinellidae, the interaction with plant leaf surface is high in all the developmental stages, both in phytophagous and in predatory species feeding on herbivorous insects. Such plant surface characteristics as wettability and roughness can have an important role in the oviposition site selection in some coccinellids, for example, in *Propylea quatuordecimpunctata* (L.) (Coleoptera: Coccinellidae) [27].

The melon ladybird beetle *Chnootriba elaterii* (Rossi, 1794) (Coleoptera: Coccinellidae) is an oligophagous multivoltine species, widespread in Eurasia, representing a serious pest of Cucurbitaceae in Southern Europe as well as in Near East, Middle East and North Africa [28,29]. The larva and adult of this species feed on leaves of pumpkin, sweet gourd, bitter gourd, cucumber, etc.; sometimes, not only leaves but also flowers or even fruits are destroyed, and seedlings of late sowings can be entirely consumed [30]. Cucurbitaceae, with ca. 800 species under 130 genera [31], are among the most economically important plant families. Most of the species are characterized by the presence of trichomes on their leaves [32]. A recent investigation demonstrated that bifid dentate claws of the adult of *C. elaterii* improve insect attachment to plant surfaces covered by trichomes such as that of *Cucurbita moschata* Duchesne ex Poir. (Cucurbitaceae) [33].

In the present study, in order to better understand the mechanical interaction between *C. elaterii* and its host plants, we measured the force exerted by adults and larvae of *C. elaterii* on leaves of different Cucurbitaceae species characterised by trichomes having different densities, lengths and diameters. Since eggs are typically laid on the abaxial side of the leaf, we tested the female attachment ability to the abaxial (lower) side of eight plant species.

## 2. Materials and Methods

### 2.1. Insects

Adults and larvae of *C. elaterii* were collected in the field around Perugia (Italy) (43.12822, 12.36097) on wild plants of *Ecballium elaterium* (L.) A. Rich (Cucurbitaceae) in September 2021. Insects were kept in a culture room under controlled conditions (25 ± 2 °C, 45 ± 15% RH, photoperiod 14L:10D) inside net cages 30 × 30 × 30 cm (Vermandel, Hulst, The Netherlands) and fed with plants of *Cucumis melo* var. inodorus Ser. (Cucurbitaceae) obtained from seeds. Eggs, larvae, and adults were maintained in separate cages. Only females and mature larvae (fourth stage) were used in the force experiments. Mated females were kept inside Petri dishes on a leaf of each tested plant species in order to have eggs laid on the abaxial side of different plant surfaces.

### 2.2. Plants

The abaxial (lower) side of the leaf blades of eight plant species from the family Cucurbitaceae was used in this study: watermelon *Citrullus lanatus* (Thunb.) Matsum. and Nakai, melon (cantaloupe) *Cucumis melo* var. cantalupensis Ser., cucumber *Cucumis sativus* L., squash or pumpkin *C. moschata*, winter squash and pumpkin, summer squash *Cucurbita pepo* L., exploding cucumber *Ecballium elaterium*, calabash *Lagenaria siceraria* (Molina) Standl. var. longissima hort., and sponge gourd *Luffa aegyptiaca* Mill. (=*L. cylindrica* M. Roem).

### 2.3. Scanning Electron Microscopy

Samples of (1) tarsi of *C. elaterii* at the adult and larval stages, (2) the abaxial sides of the tested plant leaves, and (3) eggs of *C. elaterii* laid on the different tested plant species were examined in a scanning electron microscope (SEM) Hitachi S-4800 (Hitachi High-Technologies Corp., Tokyo, Japan) equipped with a Gatan ALTO 2500 cryo-preparation system (Gatan Inc., Abingdon, UK). Plant material for cryo-SEM examination was collected from the living plants. Leaves were cut off, and 1 cm × 1 cm samples were cut out from the middle region of the leaves. Insect tarsi were dissected from insects anesthetized with carbon dioxide.

Samples were attached to a metal holder and frozen in a cryo stage preparation chamber at −140 °C. Frozen samples were sputter coated with gold-palladium (thickness 6 nm) and studied in the frozen condition (−120 °C) in the cryo-SEM at 3 kV accelerating voltage.

Description of trichomes and determination of their types were performed in accordance with [12]. Types of wax structures were identified based on [34]. Morphometrical variables of surface features were measured from digital images using SigmaScan Pro 5 software (SPSS Inc., Chicago, IL, USA). These data are presented in the text as mean ± SD for *n* = 10.

### 2.4. Light Microscopy

To visualize the real contact area between the insect tarsal attachment devices and the substrate, living females and larvae of *C. elaterii* walking on hydrophilic glass were examined with reflection contrast microscopy (RCM) using an inverted bright-field microscope ZEISS Axio Observer.A1 (Carl Zeiss Microscopy GmbH, Jena, Germany) in combination with a high-speed camera Photron FASTCAM SA1.1 (VKT Video Kommunikation GmbH—Technisches Fernsehen, Pfullingen, Germany) as described earlier [35,36,37].

### 2.5. Contact Angle Measurements

The wettability of the plant surfaces used in the experiments was characterized by measuring the contact angles of water (aqua millipore, 1 µL droplet volume) using a high-speed optical contact angle measuring device OCAH 200 (Dataphysics Instruments GmbH, Filderstadt, Germany). Sessile drop method (*C. lanatus, C. melo, C. sativus* and *C. pepo*) and sessile needle-in drop method (*C. moschata* and *L. siceraria)*, as well as the combination of both (*E. elaterium* and *L. aegyptiaca*) followed by a circle or ellipse fitting, were applied. Data are given in the text as mean ± SD for *n* = 10 measurements.

### 2.6. Force Measurements

The friction (traction) force of *C. elaterii* females on the abaxial side of the leaf blades of eight plant species from the family Cucurbitaceae and on hydrophilic glass (water contact angle of 32.49 ± 4.17° (mean ± SD)) was measured using a Biopac force tester (Biopac Systems Ltd., Goleta, CA, USA), while a centrifugal force tester was applied to measure the attachment ability of *C. elaterii* larvae to the same surfaces. Prior to the force measurements, adults and larvae were weighed on a micro-balance (Mettler Toledo AG 204 Delta Range, Greifensee, Switzerland). Experimental insects were anesthetized with CO_2_ for 60 s, and females were made incapable of flying by gluing their elytra together with a small droplet of melted bee wax. Before starting the experiments, the treated insects were left to recover for 30 min. All the experiments were performed during the daytime at 25 ± 2 °C temperature and 50 ± 5% RH.

The Biopac force tester consisted of a force sensor FORT-10 (10 g capacity; World Precision Instruments Inc., Sarasota, FL, USA) connected to a data acquisition unit MP 160 (Biopac Systems Ltd., Goleta, CA, USA). Data were recorded using AcqKnowledge 5.0 software (Biopac Systems Ltd., Goleta, CA, USA). One end of a fishing thread Gel Spun Polyethylene (Berkley Spirit Lake, IA, USA), 0.02 mm in diameter and about 10 cm long, was fixed with a droplet of molten wax to the insect elytra. The insect was attached to the force sensor by means of the thread and was allowed to move on the test substrate in a direction perpendicular to the force sensor (and parallel to the substrate). The force generated by the insect walking on hydrophilic glass and on the abaxial side of the leaves of the different plant species was measured. Insects pulled on the plant leaf in the direction from its proximal to the distal portion. Force–time curves were used to estimate the maximal pulling force produced by tethered running insects. In total, 20 females were tested on each plant species.

The centrifugal force tester [38] consists of a metal drum covered by a substrate disc to be tested. The drum is driven by a computer-controlled motor. Just above the disc, the fiber-optic sensor monitored by the computer is situated. After positioning the insect on the horizontal disc, the centrifuge drum started the rotation at a speed of 50 rev min^–1^ (0.883 rev s^−1^). The position of the insect on the drum was monitored by using a combination of a focused light beam and a fiber optical sensor. The drum speed continuously increased until the larva lost its hold on the surface under centrifugal force. The rotational speed at contact loss, the last position of *C. elaterii* larva on the drum (radius of rotation), and the larva weight were used to calculate the maximum frictional component of the attachment force. To test the larva on the abaxial side of leaves of different Cucurbitaceae species, a portion of the leaf was fixed using tape to the metal drum of the centrifuge. In order to avoid potential damage of larvae caused by numerous centrifugal experiments, 15 larvae were tested on the hydrophilic glass as a reference surface and on four of the eight plant species, and the other 15 larvae were tested on glass and on four remaining plant species. For each larva, different plant species were tested in random order.

### 2.7. Statistical Analysis

The normalized friction force (the ratio between the friction force on leaves and the friction force on the glass of each insect individual) of *C. elaterii* females on different leaf surfaces, the normalized friction force obtained in *C. elaterii* larvae, the density of glandular and non-glandular trichomes as well as the length and diameter of non-glandular trichomes in the different plant species were analyzed using one-way analysis of variance (ANOVA), followed by the Tukey unequal N HSD post hoc test for multiple comparisons between means (Statistica 6.0, Statsoft Inc. 2001, Tulsa, OK, USA). Before the parametric analysis, all the data were subjected to Box-Cox transformations in order to reduce data heteroscedasticity [39].

## 3. Results

### 3.1. Adult and Larval Attachment Devices

**Figure 1:**
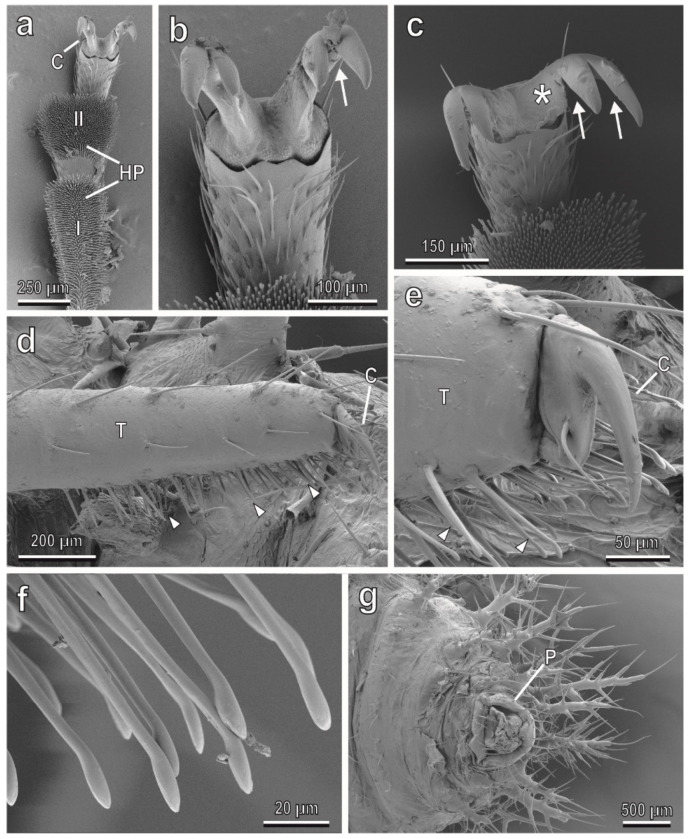
Tarsi of the female (**a**–**c**) and of the larva (**d**–**g**) of *Chnootriba elaterii*, cryo-SEM. (**a**) Hairy pads (HP) covered with numerous tenent setae located on the ventral side of the first (I) and second (II) tarsal segments. C, claws. (**b**,**c**) Details of the bifid claws with a basal tooth (asterisk). Note the deep clefts (arrows). (**d**,**e**) Single pretarsal claw (C) and tarsal tenent setae (arrowheads) located on the tarsus (T). (**f**) Distal portions of tenent setae. (**g**) Detail of the pygopodium (P) at the end of the abdomen.

The tarsi in the female of *C. elaterii* are composed of four tarsal segments. The tarsal attachment organs consist of a pair of pretarsal claws and two hairy pads located on the ventral side of the first and second tarsal segments. The pads are covered with numerous “tenent setae” (hairs modified in the distal portion to increase the actual area of attachment to the surface) (Figure 1a). Each claw is bifid with a basal tooth separated from the claw by a deep cleft (Figure 1b,c). This kind of claw is called bifid dentate or bifid appendiculate.

**Figure 2:**
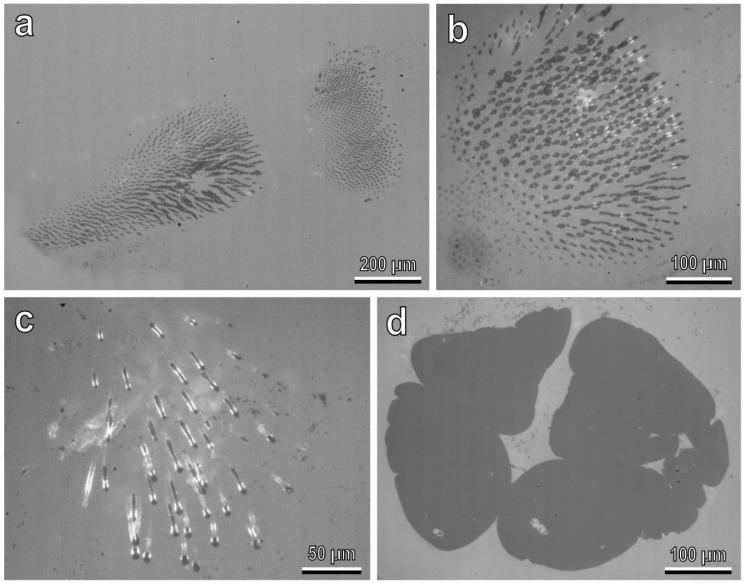
Footprints of females (**a**,**b**) and larvae (**c**,**d**) walking on hydrophilic glass, visualized with reflection contrast microscopy and a high-speed camera. The areas of contact between attachment structures and glass appear dark. (**a**,**b**) Contact area of tenent setae of adhesive pads. (**c**) Contact area of tarsal tenent setae. (**d**) Contact area of pygopodium.

The attachment devices in larvae of all four instars are constituted of a single pretarsal claw (Figure 1d,e), tenent setae located on the tarsus (Figure 1d–f) and a pygopodium or “anal organ” at the end of the abdomen (Figure 1g). A widened end plate (spatula) is present in the distal portion of a tenent seta (Figure 1f). Footprints of females walking on hydrophilic glass (Figure 2a,b) reveal that in both the first and second tarsal segments, only distal portions of each pad are in contact with the substrate, whereas larval prints show that both tarsal tenent setae and the pygopodium are responsible for attachment to smooth surfaces (Figure 2c,d).

### 3.2. Characterization of Plant Surfaces

**Figure 3:**
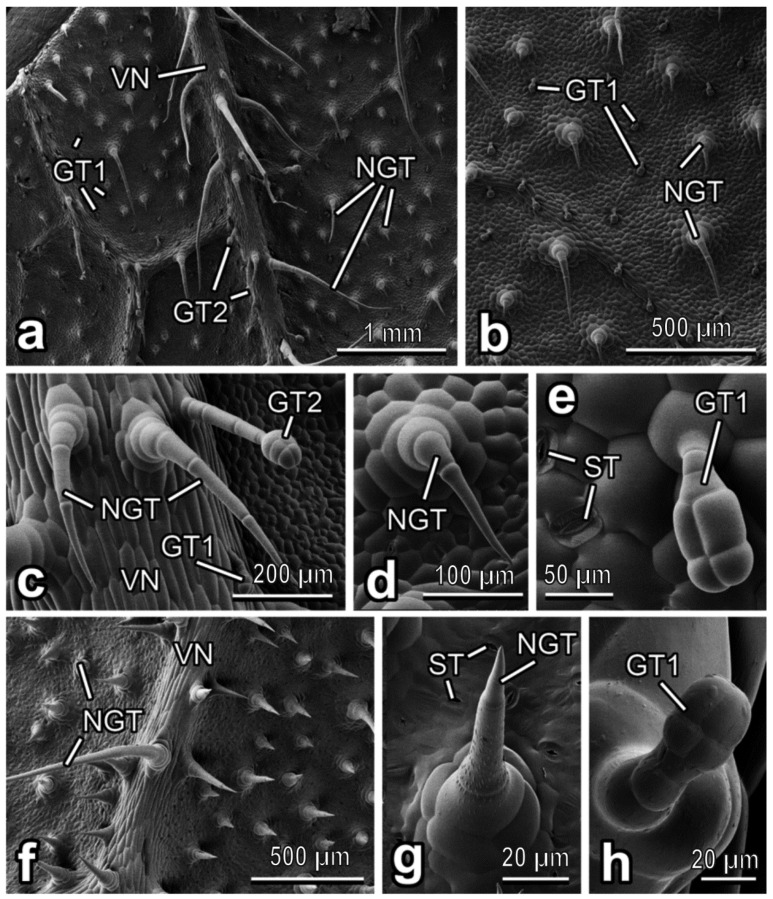
Abaxial leaf side of *Citrullus lanatus* (**a**–**e**) and *Cucumis melo* (**f**–**h**), cryo-SEM. (**a**,**f**) Leaf surface with the prominent vein(s). (**b**) Leaf surface between the prominent veins. (**c**) The vein bearing both non-glandular trichomes and a glandular (long-stalked) one. (**d**,**g**) Non-glandular trichome from the leaf region between the veins. (**e**,**h**) Glandular (short-stalked) trichome. Abbreviations: GT1: clavate short-stalked glandular trichome; GT2: capitate long-stalked glandular trichome; NGT: non-glandular trichome; ST: stoma; VN: vein.

In *C. lanatus*, the abaxial leaf side bears both non-glandular and glandular trichomes, which are regularly distributed over the entire leaf surface (Figure 3a–c). The non-glandular trichomes are multicellular and uniseriate, with big multicellular sockets at the base (Figure 3c,d). They are non-branched, have cone shapes and very sharp tips. These trichomes are bent or curved and accumbent, especially those situated between the leaf veins (Figure 3b,d). On the veins, the non-glandular trichomes are more squarrose being pointed to different directions (Figure 3a,c) and much longer than those between the veins. In the areas between the veins, glandular trichomes are clavate, short-stalked, with the four-celled head region (“characteristic” short-stalked *Cucurbita* type or type I according to [40] (Figure 3e). They are smaller (length: 51.50 ± 10.11 µm) than non-glandular ones and densely cover the surface (abundance: ca. 15 mm^−2^). The glandular trichomes situated on the veins are capitate, long-stalked, with two- or often also multi(four)-celled heads and a distinct “neck” region. They are much longer (length: 259.66 ± 82.07 µm) than the glandular short-stalked trichomes. With the only difference in the number of head cells (two and more vs. two or three), these trichomes resemble the long-stalked neck-cell type or type II described in *C. pepo* var. *styriaca* [40]. Moreover, plentiful stomata are scattered (abundance: up to ca. 500 mm^−2^) over the leaf surface (Figure 3e).

The abaxial leaf side in *C. melo* bears numerous, regularly distributed non-glandular trichomes, which are responsible for uniform hairy coverage of the surface (Figure 3f). They are multicellular, uniseriate and sit on rather high multicellular sockets (Figure 3g). These trichomes are non-branched, cone-shaped, with slightly more narrow apical regions and very sharp tips (Figure 3f,g). They are usually straight or gently inclined; on the veins, they are bent and pointed in different directions, being longer here (Figure 3f). The trichome surface, except for the most apical cell, is clearly microstructured with round and ellipsoid knobby protrusions (Figure 3g). Both types of glandular trichomes, such as (1) clavate short-stalked, with four-celled heads region (type I [40]) (ca. 70 µm long) (Figure 3h) and (2) capitate long-stalked with multi(four)-celled heads and distinct “neck” region similar to those found in *C. lanatus* (see above) resembling type II [40] (ca. 120 µm long), occur extremely sparsely (almost solitarily). Plenty of stomata (abundance: ˃300 mm^−2^) are regularly dispersed over the surface (Figure 3g).

**Figure 4:**
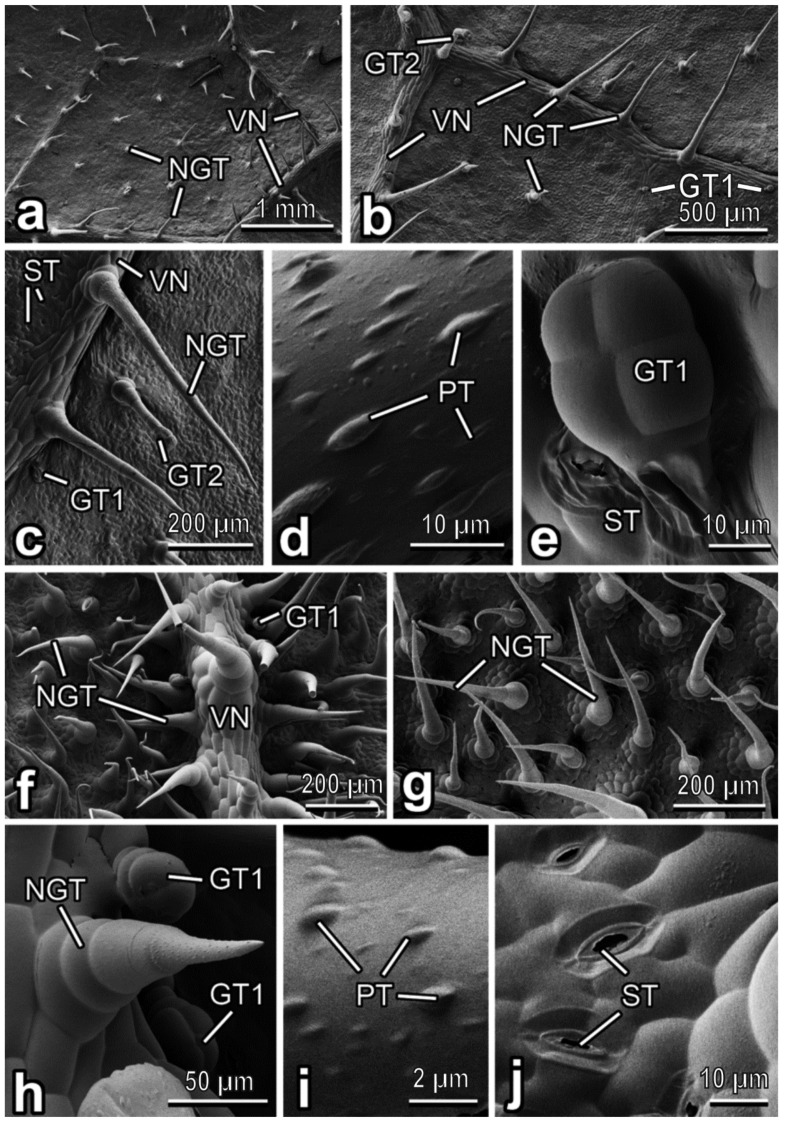
Abaxial leaf side of *Cucumis sativus* (**a**–**e**) and *Cucurbita moschata* (**f**–**j**), cryo-SEM. (**a**) General view of the leaf surface. (**b**,**c**,**f**) Leaf surface with the vein(s). (**d**,**i**) Microsculptured surface of the non-glandular trichome. (**e**) Glandular (short-stalked) trichome. (**g**) Leaf surface between the veins. (**h**) The vein bearing both non-glandular and glandular (short-stalked) trichomes. (**j**) Stomata. Abbreviations: GT1: clavate short-stalked glandular trichome; GT2: capitate long-stalked glandular trichome; NGT: non-glandular trichome; PT: protrusion; ST: stoma; VN: vein.

In *C. sativus*, the abaxial side of the leaf blade bears relatively sparse trichome coverage (Figure 4a). Both non-glandular and glandular trichomes of two types are uniformly distributed over the entire leaf surface (Figure 4b,c). Non glandular trichomes are more abundant than glandular ones. These multicellular, uniseriate, conical, non-branched trichomes have rather small multicellular bases and sharp tips (Figure 4b,c). Their surface is microsculptured with plentiful, very prominent rounded/ellipsoid knobby protrusions (Figure 4d). Non-glandular trichomes are straight or slightly bent between the veins but usually bent or curved and pointed in various directions on the veins (Figure 4a–c). Clavate short-stalked glandular trichomes with four-celled heads (type I [40]) are small (length: 50.81 ± 3.55 µm) and tightly accumbent to the surface (Figure 4b,c,e), whereas capitate long-stalked ones with usually four-celled heads and typical “neck” region (similar to those in *C. lanatus* and resembling type II [40]) are much longer (ca. 200–250 µm long), straight or somewhat bent and occur significantly more rare (Figure 4b,c). Stomata (Figure 4c,e) are present in a high number (abundance: ˃700 mm^−2^).

The abaxial leaf surface of *C. moschata* is regularly covered by both non-glandular and glandular trichomes situated on both leaf veins and areas between the veins (Figure 4f–h). Whereas plentiful non-glandular trichomes are distributed almost equally on both the veins and between them (Figure 4f–h), scanty glandular trichomes are located mostly in the veins (Figure 4f,h). The non-glandular trichomes are multicellular, uniseriate, with very prominent multicellular sockets, non-branched, cone-shaped, with rather sharp tips and microsculptured surfaces bearing numerous ellipsoid knobby protrusions (Figure 4h,i). These trichomes are either straight or, more often, curved, usually slightly inclined or bent and create relatively uniform and dense leaf pubescence (Figure 4f–h). Glandular clavate, short-stalked trichomes with a four-celled head region (type I [40]) are much smaller (length: 36.27 ± 26.07 µm) than non-glandular ones and occur very sparsely (Figure 4f,h). Numerous (abundance: ˃250 mm^−2^) stomata are regularly distributed over the abaxial leaf side (Figure 4j).

**Figure 5:**
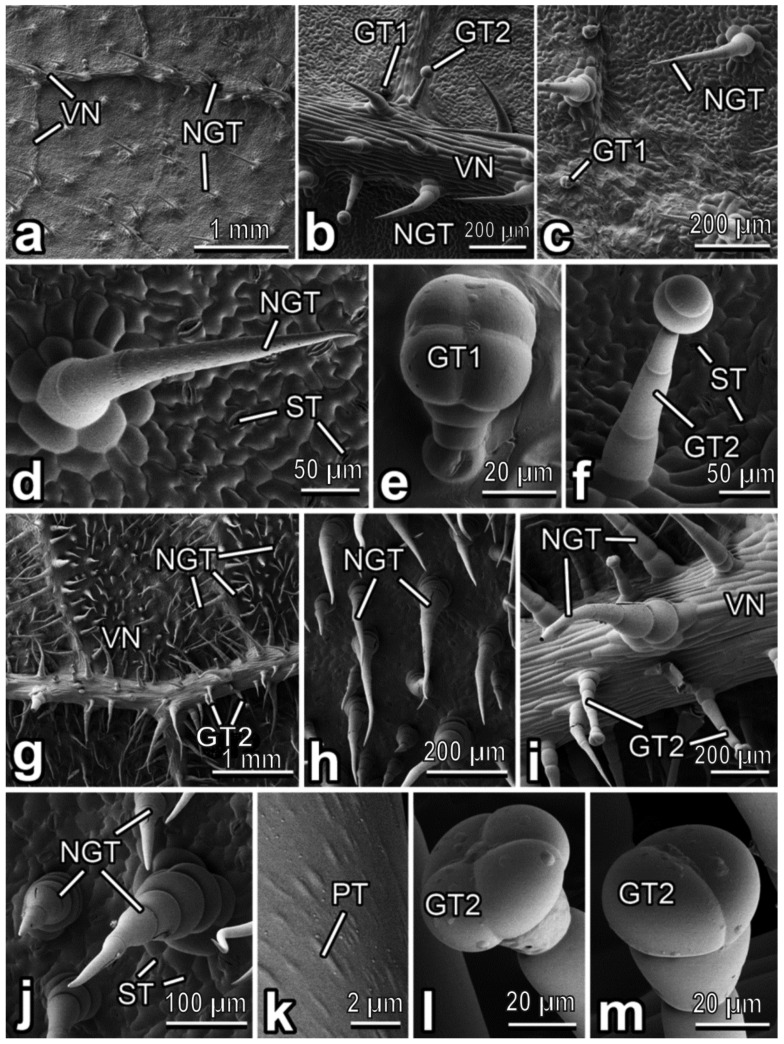
Abaxial leaf side of *Cucurbita pepo* (**a**–**f**) and *Ecballium elaterium* (**g**–**m**), cryo-SEM. (**a**,**g**) General view of the surface. (**b**,**i**) Leaf surface with the vein. (**c**,**h**) Leaf surface between the veins. (**d**,**j**) Non-glandular trichome. (**e**) Glandular (short-stalked) trichome. (**f**,**l**,**m**) Glandular (long-stalked) trichome. (**k**) Microsculptured surface of the non-glandular trichome. Abbreviations: GT1: clavate short-stalked glandular trichome; GT2: capitate long-stalked glandular trichome; NGT: non-glandular trichome; PT: protrusion; ST: stoma; VN: vein.

The *C. pepo* leaf is noticeably pubescent on its abaxial side, on both the veins and between them (Figure 5a). The uniform hairy coverage is composed of both non-glandular trichomes and glandular ones of two types (Figure 5b,c). Regularly distributed multicellular, uniseriate, non-branched, cone-shaped non-glandular trichomes have multicellular sockets, sharp tips and the surface microsculptured with numerous knobby protrusions of various, but more often elongated shapes (Figure 5d). They are either curved or bent and usually pointed in one preferred direction (Figure 5a–d). Glandular clavate, short-stalked trichomes with four-celled heads (type I [40]) are the smallest among all trichomes (length: 49.88 ± 1.91 µm) (Figure 5e). They are tightly accumbent to the surface and rather sparsely distributed (Figure 5b,c,e). Capitate long-stalked glandular trichomes with typical two-celled heads and slim “necks” (type II [40]) are much longer (length: 191.55 ± 23.48 µm) (Figure 5f) and occur more often than clavate, short-stalked trichomes (Figure 5b,c). Moreover, numerous (abundance: ca. 350 mm^−2^) stomata are located on the *C. pepo* abaxial leaf surface (Figure 5d,f).

The *E. elaterium* abaxial leaf surface has a wooly appearance because of a trichome coverage created in the main by non-glandular trichomes pointed in different directions (Figure 5g). These densely and uniformly occurring multicellular, uniseriate, non-branched, cone-shaped trichomes have rather low either uni- or multicellular sockets (Figure 5h–j). The cells composing the trichome are much wider in their basal part and become narrower till a very sharp and flexible tip (Figure 5j). Since these cells, except the distal one, have a barrel-like shape, boundaries between the cells are clearly seen. The trichome surface is covered with numerous knobby ellipsoid or elongated protrusions (Figure 5k). On the veins, non-glandular trichomes are large than those located between the veins (Figure 5g). Moreover, glandular trichomes are sparsely located mostly in the veins (Figure 5i). They are capitate, with long multicellular stalks (looking like the central part of non-glandular trichomes in this plant species) (length: 249.83 ± 22.03 µm), distinct “necks”, and two- or four-celled heads (Figure 5i,l,m). These trichomes resemble, to some extent, the long-stalked glandular trichomes found in *C. lanatus* (see above) and type II in *C. pepo* var. *styriaca* [40]; however, also typical type II trichomes [40] are also present. Plentiful (abundance: ˃240 mm^−2^) stomata are spread over the surface (Figure 5j).

**Figure 6:**
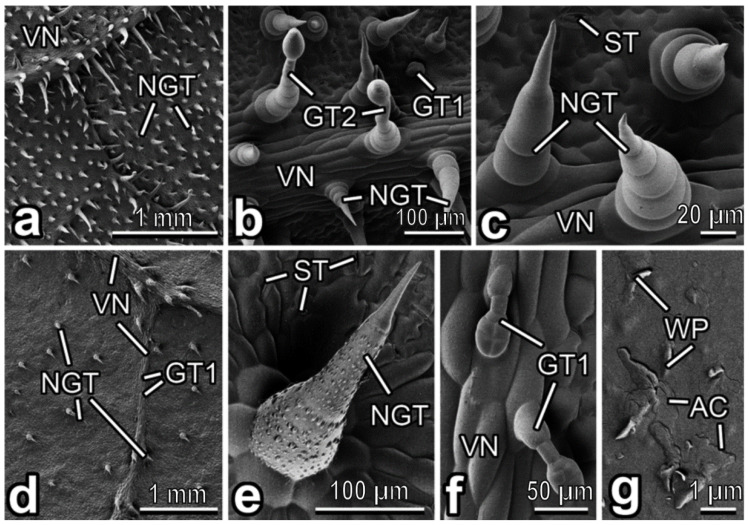
Abaxial leaf side of *Lagenaria siceraria* (**a**–**c**) and *Luffa aegyptiaca* (**d**–**g**), cryo-SEM. (**a**,**d**) General view of the surface. (**b**) Leaf surface with the vein. (**c**,**e**) Non-glandular trichome(s). (**f**) Glandular (short-stalked) trichomes. (**g**) Epicuticular wax coverage. AC; artificial cracks in the smooth epicuticular wax layer; GT1: clavate short-stalked glandular trichome; GT2: capitate long-stalked glandular trichome; NGT: non-glandular trichome; ST: stoma; VN: vein; WP: epicuticular wax projection.

The dense pubescence of the abaxial leaf side in *L. siceraria* consists of numerous non-glandular trichomes distributed rather regularly over the surface, whereas glandular trichomes are located on and near the veins (Figure 6a,b). Non-glandular trichomes are very similar to those found in *E. elaterium*, with usually simple unicellular sockets (Figure 6c). Glandular trichomes of both types, (1) small (length: 51.06 ± 8.61 µm) clavate short-stalked ones with four-celled heads (see the description in *C. lanatus*) and (2) long (length: 118.40 ± 56.20 µm) capitate long-stalked ones with typical both necks and two-celled heads, exactly correspond to types I and II described in *C. pepo* var. *styriaca* [40]. Moreover, stomata in a great number (abundance: ca. 240 mm^−2^) are present on the surface (Figure 6c).

In *L. aegyptiaca*, the abaxial leaf side bears sparsely but regularly distributed, rather short non-glandular and glandular trichomes (Figure 6d). The non-glandular trichomes are multicellular, uniseriate, non-branched and cone-shaped, with relatively short multicellular sockets and sharp tips (Figure 6e). The microsculpturing (knobby rounded/ellipsoid protrusions) of the trichome surface is very prominent in its basal and central parts but is completely lacking in the distal cell (Figure 6e). Clavate short-stalked glandular trichomes (Figure 6f) are somewhat shorter (length: 79.91 ± 13.09 µm) than non-glandular ones. The heads are mostly four-celled (typical type I [40]) (Figure 6f), while also multicellular heads are present in very few cases. Numerous (abundance: ca. 200 mm^−2^) stomata are regularly located on the surface between the trichomes (Figure 6e). The cuticle is noticeably covered with a smooth layer of epicuticular wax (detected in SEM due to artificial cracking [40]) and bears solitarily dispersed, microscopic (length: 0.72 ± 0.42 µm), scale-like epicuticular wax projections (Figure 6g).

**Figure 7:**
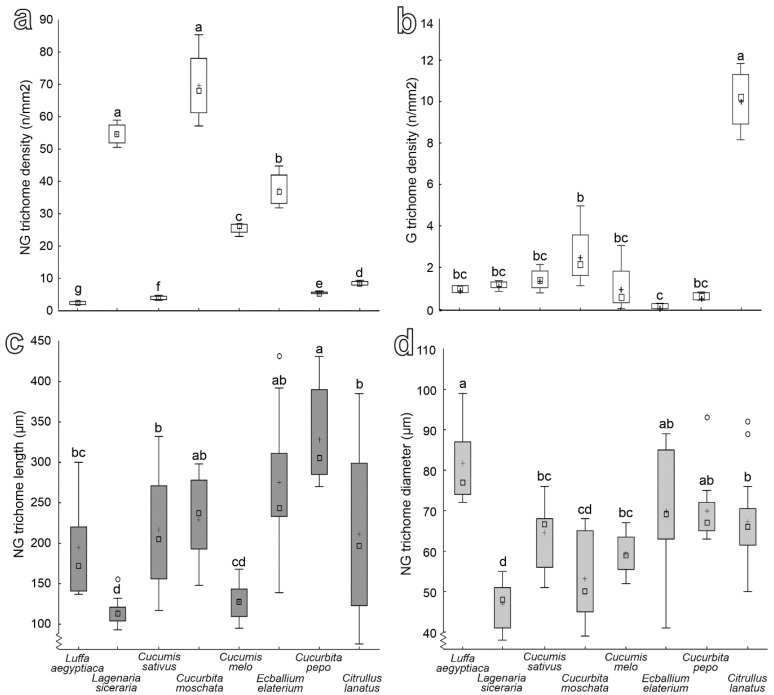
Density of non-glandular (**a**) and glandular trichomes (**b**) and length (**c**) and diameter (**d**) of non-glandular trichomes in the different Cucurbitaceae plant species. Boxplots show the interquartile range and the medians; whiskers indicate 1.5 × interquartile range, “°” is outlier and “+” shows the mean. Boxplots with different letters are significantly different at *p* < 0.05 (Tukey unequal N HSD post hoc test, one-way ANOVA).

Data on the density of non-glandular and glandular trichomes and of the length and diameter of the non-glandular trichomes for the studied plant species are presented in Figure 7. In particular, non-glandular trichomes density is significantly higher in both *L. siceraria* and *C. moschata* than in the other species. The latter species show a progressive decrease of non-glandular trichomes density in the following order: *E. elaterium*, *C. melo*, *C. lanatus*, *C. pepo*, *C. sativus*, *L. cylindrica* (Figure 7a) (*F* = 383.51; d.f. = 7, 23; *p* < 0.0001). Glandular trichomes density is the highest in *C. lanatus* and the lowest in *E. elaterium*, while it is intermediate in the other plant species (Figure 7b) (*F* = 42.84; d.f. = 7, 23; *p* < 0.0001). Non-glandular trichomes are significantly longer in *C. pepo*, shorter in both *C. melo* and *L. siceraria*, and have an intermediate length in *E. elaterium, C. moschata, C. sativus, C. lanatus* and *L. cylindrical* (Figure 7c) (*F* = 12.08; d.f. = 7, 85; *p* < 0.0001). Non-glandular trichome diameter is the largest in *L. cylindrica* and the smallest in *L. siceraria.* In the remaining plant species, it is intermediate (Figure 7d) (*F* = 13.64; d.f. = 7, 85; *p* < 0.0001).

All plant surfaces studied were relatively unwettable (contact angle ˃ 90°) by water, being either hydrophobic in *C. sativus* (99.5 ± 9.4°), *C. pepo* (99.8 ± 6.0°), *C. lanatus* (102.6 ± 4.8°), *C. melo* (103.5 ± 4.7°) and *L. aegyptiaca* (119.4 ± 12.0°) or even highly hydrophobic in *E. elaterium* (135.4 ± 9.6°), *L. siceraria* (143.6 ± 7.6°) and *C. moschata* (147.5 ± 5.1°).

### 3.3. Adult and Larval Attachment Ability to Different Cucurbitaceae Species

**Figure 8:**
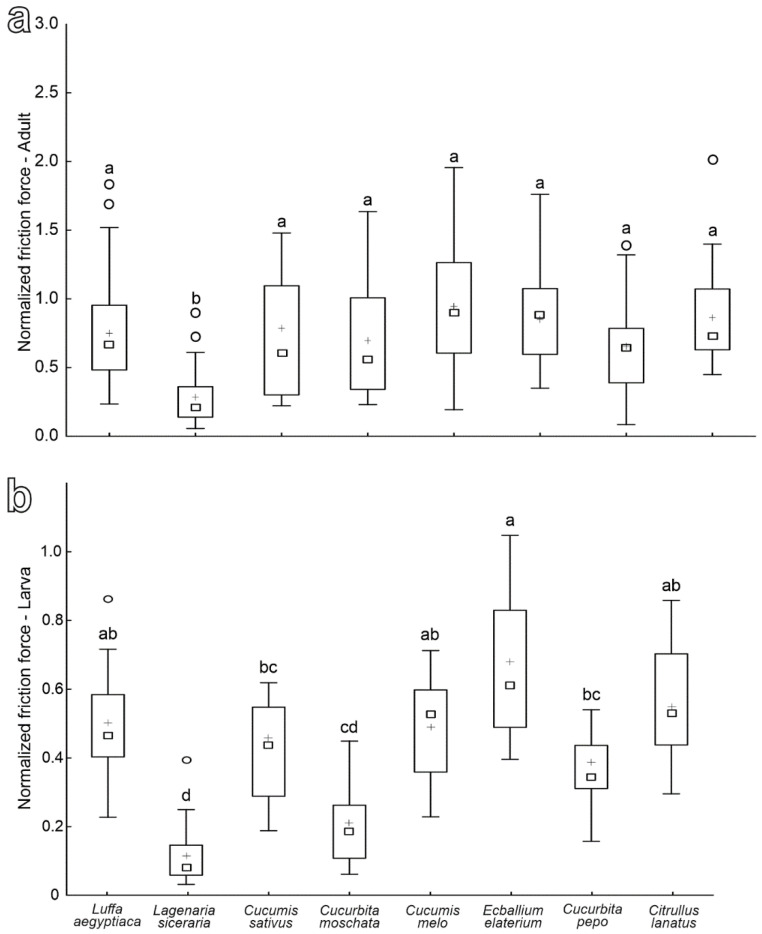
Normalized friction force of females (**a**) and larvae (**b**) of *Chnootriba elaterii* on the abaxial leaf side of different Cucurbitaceae species. Boxplots show the interquartile range and the medians, whiskers indicate 1.5× interquartile range, “°” show the outliers and “+“ show the means. Boxplots with different letters are significantly different at *p* < 0.05 (Tukey unequal N HSD post hoc test, one-way ANOVA).

The normalized friction force produced by females on leaves of the different plant species is similar on all tested species except for *L. siceraria*, where it is significantly reduced (Figure 8a) (*F* = 7.84; d.f. = 7, 153; *p* < 0.0001).

The values of the normalized friction force registered in larvae on different plant species are the highest on *E. elaterium*, *L. aegyptiaca*, *C. melo* and *C. lanatus* leaves, the lowest on both *L. siceraria* and *C. moschata* and intermediate on the other tested plant species (*C. sativus* and *C. pepo*) (Figure 8b) (*F* = 12.01; d.f. = 7, 111; *p* < 0.0001).

The mean friction force of females on hydrophilic glass is 9.77 ± 0.56 mN, while that of larvae reaches 30.59 ± 1.61 mN. As for the mean safety factors on hydrophilic glass, it is 14.73 ± 1.07 in females and rises to 57.48 ± 3.08 in larvae.

### 3.4. Egg-plant Surface Interaction

**Figure 9:**
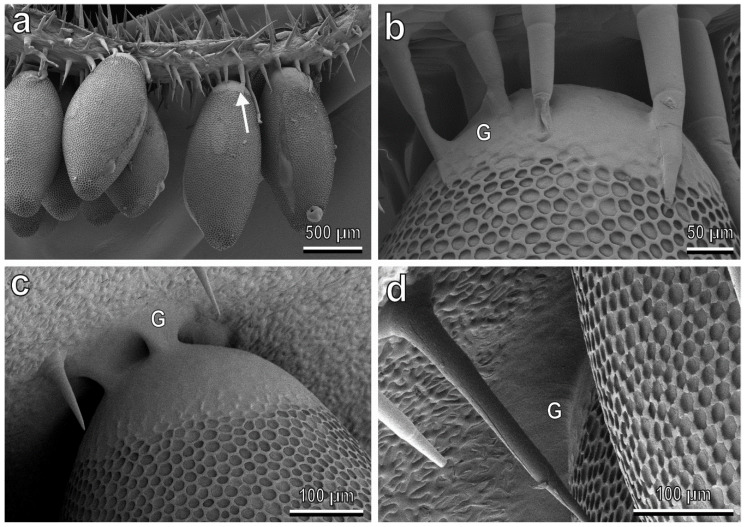
*Chnootriba elaterii* eggs laid on the abaxial side of *Lagenaria siceraria* (**a**,**b**) and *Cucumis sativus* (**c**,**d**) leaves, cryo-SEM. (**a**,**b**) Densely situated trichomes (arrow) do not allow the egg glue (G) to reach the leaf surface. (**c**,**d**) The egg glue (G) adheres well to the leaf surface, thus replicating the leaf surface microsculpture.

The cryo-SEM analysis of the interface between the base of *C. elaterii* eggs and the abaxial side of leaves of different plant species (Figure 9) shows that in the plant species with a dense pubescence such as *L. siceraria* (Figure 9a,b), the glue cannot reach the leaf surface and the trichomes keep the egg away from the leaf surface. In plants with a low trichomes abundance, such as *C. sativus,* the glue readily spreads over the leaf surface (Figure 9c,d).

## 4. Discussion

Leaves of plant species belonging to Cucurbitaceae are covered by glandular and non-glandular trichomes of different shapes and sizes, whose features can be important for taxonomic purposes [41]. The data collected in the present investigation showed that glandular trichomes have much lower density compared with non-glandular trichomes in all tested plant species except *C. lanatus,* where the density of glandular trichomes is considerably higher than in other tested plants and similar to that of non-glandular ones in this species. However, a high number of glandular trichomes in *C. lanatus* seems not to affect the attachment ability of *C. elaterii* at both adult and larval stages: our friction force experiments showed that the insect attachment ability to leaves of this plant was rather good and not significantly lower than to leaves of other species. This result differs from what was observed for predatory coccinellids, such as *Hippodamia convergens* Guérin-Méneville (Coleoptera: Coccinellidae), in which all instars were affected by the sticky exudates of leaf trichomes in tobacco cultivars [42]. This aspect could be related to (1) different amounts or different chemical compositions of the glandular secretion in these two plant species or (2) special adaptation of *C. elaterii* to the life in the vicinity of glandular trichomes of its host plants. For example, the cuticle of the mirid bug *Pameridea roridulae* Reuter (Heteroptera: Miridae) living on the plant *Roridula gorgonias* Planch. (Roridulaceae), which is rich in adhesive glandular trichomes, bears an epicuticular greasy secretion preventing the formation of contacts between the sticky plant secretion and the solid insect cuticle [43]. Further investigations on *C. elaterii* are needed to clarify this issue.

The attachment forces of the *C. elaterii* adults to different tested plant species were always lower than those recorded on hydrophilic glass. This result can be explained not only by the presence of trichomes, which did not allow the tenent setae of the hairy pads to reach the leaf surface but also by the rather hydrophobic properties of leaves in all tested Cucurbitaceae species. Indeed, for many insect species at both adult and larval stages, a higher attachment ability on hydrophilic smooth surfaces in comparison with smooth hydrophobic ones has been previously observed [27,44,45,46,47,48,49,50,51,52,53,54,55].

As for the influence of non-glandular trichomes on the mechanical interaction between the ladybird and the plants, female attachment ability was similar on all the tested plants except *L. siceraria*. The attachment to the leaf of this plant was particularly challenging for both the adults and larvae of *C. elaterii*. This can be explained by the combination of a high density of trichomes and their small length and diameter. Moreover, the cells surrounding the bases of the trichome shafts in *L. siceraria* are not enriched by silicon or calcium, differently from other Cucurbitaceae, such as *C. sativus*, *C. melo,* and *C. lanatus,* which show silicon or calcium in this area [56]. For this reason, *L. siceraria* trichomes tend to bend at the base [56]. The above features are responsible for a “velvet like appearance” of the leaf surface in this plant species, which can cause difficulty for an insect when it tries to adhere to this leaf. Our results are in agreement with the data of the previous study testing the friction force of adults in different Coleoptera species, such as *C. elaterii*, *Harmonia axyridis* Pallas (Coccinellidae) and *Chrysolina herbacea* Duftschmid (Chrysomelidae) on a *C. moschata* leaf and its resin replica having higher trichome stiffness [33]. Those experiments revealed that insect attachment ability increased with an increase in trichome stiffness.

Recent investigations on the chemical interaction between *C. elaterii* and the same Cucurbitaceae plant species tested here showed that this insect can perceive the volatile chemicals (VOCs) produced by *L. siceraria*. The electroantennographic study showed that the antennae of *C. elaterii* adults clearly responded to the extract of this plant [57]. At the same time, if females could choose between leaves of *L. siceraria* and other Cucurbitaceae, such as *C. melo*, *C. sativus*, *C. pepo*, *C. moschata* and *C. lanatus*, they never chose *L. siceraria* [58]. Such behavior could be caused by plant chemical defenses (we cannot exclude the presence of gustatory cues on the leaf reducing adult attraction) but also by plant mechanical defenses represented by short, dense and flexible trichomes. Indeed, when adults choose a place for oviposition, plant mechanical features can be relevant. In this regard, it is important to note that a good attachment to the leaf surface is a fundamental prerequisite for oviposition behavior since Coccinellidae species typically lay their eggs upside down on the abaxial leaf surface. In agreement with this, in the whitefly predator ladybird *Serangium japonicum* Chapin (Coleoptera: Coccinellidae), a positive correlation between the female oviposition preference and the attachment force of adults on the plant leaf has been recently demonstrated [59].

A firm attachment of an egg to a plant surface is fundamental for larval survival in a phytophagous species. Leaf trichomes can reduce coccinellid egg adhesion compared to smooth leaf surfaces, as demonstrated for the eggs of *H. axyridis* and *P. quatuordecimpunctata* laid on the stellate trichomes of *S. melongena* [27]. Our cryo-SEM observations revealed that the dense trichome coverage on the leaf of *L. siceraria* reduced the contact of the egg glue with the epidermal leaf surface, thus highly affecting the egg attachment ability to the leaf compared with plants having a rather low density of trichomes, like *C. sativus*, where the egg glue can reach the leaf surface. These results highlight the importance of plant mechanical barriers, which can have a strong impact on shaping the functional composition of the herbivore assemblage [60].

In our experiments, females of *C. elaterii* performed well on all the tested species (except *L. siceraria)*, even on *C. moschata* showing a high density of trichomes that are longer, wider and stiffer than those in *L. siceraria*. Such an ability to attach to hairy plant surfaces can be related to the presence of bifid dentate claws with deep clefts, which can interlock with trichomes, as recently demonstrated [33]. In agreement with this, our data demonstrated that the larvae of *C. elaterii* having a single claw cannot adhere to *C. moschata* as well as the adults: larval attachment ability to *C. moschata* was low and not significantly different from that recorded on *L. siceraria*. For the larvae, a dense layer of trichomes (long or short, stiff or flexible) is challenging in any case. This observation confirms that the trichome effect can differ according to the insect developing stage, as already shown in lepidopterous defoliators, where the pubescence of soybean functions as a resistance mechanism to the larval stage, but enhances adult oviposition when compared to plants without pubescence [61].

In conclusion, the data presented here deepen the knowledge of the mechanical interaction of *C. elaterii* at different developmental stages with different Cucurbitaceae species. Such data, combined with the information on the chemical and visual cues involved in the host plant choice, can help to unravel the complex factors driving the coevolution between an oligophagous insect and its host plant species. Moreover, identifying the chemical cues involved in the host plant selection and the morphological and leaf traits of Cucurbitaceae associated with their resistance against phytophagous insects can help to develop environmentally friendly pest control methods.

## Data Availability

The data presented in this study are openly available in Mendeley Data at https://data.mendeley.com/datasets/mnpv3mmftz/1 (accessed on 20 October 2022).
